# Towards the isolation and estimation of elemental carbon in atmospheric aerosols using supercritical fluid extraction and thermo-optical analysis

**DOI:** 10.1007/s00216-017-0380-0

**Published:** 2017-05-08

**Authors:** Hafiz Abdul Azeem, Johan Martinsson, Kristina Eriksson Stenström, Erik Swietlicki, Margareta Sandahl

**Affiliations:** 1 Centre for Analysis and Synthesis, Department of Chemistry, Lund University, Naturvetarvägen 14/Sölvegatan 39 A-C, 22100 Lund, Sweden; 2 Division of Nuclear Physics, Department of Physics, Lund University, Professorsgatan 1, 22100 Lund, Sweden; 3 Centre for Environmental and Climate Research, Lund University, Sölvegatan 37, 22362 Lund, Sweden

**Keywords:** Atmospheric aerosols, Elemental carbon, Pyrolytic organic carbon, Source apportionment, Supercritical carbon dioxide, Thermo-optical analysis

## Abstract

Air-starved combustion of biomass and fossil fuels releases aerosols, including airborne carbonaceous particles, causing negative climatic and health effects. Radiocarbon analysis of the elemental carbon (EC) fraction can help apportion sources of its emission, which is greatly constrained by the challenges in isolation of EC from organic compounds in atmospheric aerosols. The isolation of EC using thermo-optical analysis is however biased by the presence of interfering compounds that undergo pyrolysis during the analysis. EC is considered insoluble in all acidic, basic, and organic solvents. Based on the property of insolubility, a sample preparation method using supercritical CO_2_ and methanol as co-solvent was developed to remove interfering organic compounds. The efficiency of the method was studied by varying the density of supercritical carbon dioxide by means of temperature and pressure and by varying the methanol content. Supercritical CO_2_ with 10% methanol by volume at a temperature of 60 °C, a pressure of 350 bar and 20 min static mode extraction were found to be the most suitable conditions for the removal of 59 ± 3% organic carbon, including compounds responsible for pyrolysis with 78 ± 16% EC recovery. The results indicate that the method has potential for the estimation and isolation of EC from OC for subsequent analysis methods and source apportionment studies.

## Introduction

Atmospheric aerosols are known to have negative effects on human health. Exposure to aerosols is one of the major causes of lung diseases and cardiovascular morbidity and mortality in the world [1, 2]. Aerosols also contribute to climate change due to their light scattering/absorbing properties and ability to promote cloud formation [3]. A significant fraction of atmospheric aerosols consists of carbonaceous matter [4] with up to 25% of total mass of PM_10_ in Europe [5]. Furthermore, combustion of biomass and fossil fuels are among the most prominent sources of emissions of organic aerosols [6]. Generally, aerosols resulting from combustion are categorized into soot, here presented as elemental carbon (EC), and organic carbon (OC) with some controversies. Following Petzold et al. [7] and Lack et al. [8], we use the term EC when describing the thermally stable carbonaceous aerosols (≈4000 K) that can be oxidized at >340 °C in an oxidative environment. Incomplete combustion leads to the formation of EC that contains structures like graphite, carbon nanospheres, multiple aromatic layers, and chars with low H/C and O/C ratios [9–11]. Some huge three-dimensional organic polymers may also be regarded as EC [12, 13]. OC is a combination of organic compounds with smaller masses, e.g., non-volatile hydrocarbons, organic acids, and anhydrous sugars. OC originates from both biogenic and anthropogenic sources, whereas EC originates mainly from anthropogenic sources including combustion of biomass and fossil fuels. Hence, EC is considered as an emission marker for biomass and fossil fuel combustion produced in air starved, high temperature combustion conditions. Unfortunately, the relative contribution of anthropogenic emissions from combustion of biomass and fossil fuels is not very well known. We want to contribute filling the knowledge gaps regarding the contribution of different sources of combustion to anthropogenic carbonaceous aerosols. Estimation of OC and EC can provide valuable information about the role of different emission sources. Furthermore, radiocarbon (^14^C) analysis of isolated EC can be used to discriminate between aerosols originating from biomass combustion and from fossil fuel combustion. The radiocarbon analysis takes advantage of the fact that fossil fuels are completely free from ^14^C, while modern biomass contains a known amount of naturally produced ^14^C as well as ^14^C resulting from atmospheric nuclear tests in the twentieth century [6, 14–23]. This knowledge on relative contribution of emission sources might be valuable for stakeholders and policymakers in order to make scientifically sound decisions regarding mitigation of anthropogenic emissions of EC, taking both climate and health into consideration.

The most commonly used methods of analysis for EC are based on the principle of evolved gas analysis, also referred to as thermal and thermo-optical analysis (TOA). Raman spectroscopy (RS) and the insolubility method proposed by Lack et al. [8] are two other common methods. The TOA and RS methods have been developed on the basis of thermal and optical properties of OC and EC. Insolubility methods are based on the most widely accepted assumption that EC is insoluble in acids, bases, and organic solvents at room temperature [8, 7, 24]. The analysis methods are used to derive EC as mass units in aerosol filter samples. TOA is simple and chemical standards can be used for calibration but there is no generally accepted method for calibration to atmospheric EC and the results are subject to bias due to the pyrolysis of organic compounds and interfering inorganic substances. RS provides molecular light scattering information based on vibrational and other low-frequency modes [25] that can be used for qualitative as well as quantitative analysis [26, 27]. RS spectra and derived EC can be calibrated with commercially available material; however, co-emitting organic compounds may lead to bias. Scarce information is available on insolubility method in terms of uncertainty, calibration, and bias. The sample treatment may take several hours [8], sometimes overnight [9].

Isolation of EC by the removal of OC is also challenging. Previous studies have connected the insolubility method to thermal methods in order to isolate EC from OC prior to ^14^C analysis [28, 9, 29]. In these studies, the filter samples were pretreated with ultrapure water for the removal of water-soluble organic compounds followed by thermal methods. Wet sample pretreatment methods including use of water [30, 31] and organic solvents [32] were also investigated to overcome bias induced by interfering organic compounds responsible for pyrolysis in TOA. Cavalli et al. [30] and Piazzalunga et al. [31] investigated water pretreatment of ambient aerosol samples followed by TOA. Cheng et al. [32] removed 55% OC in ambient aerosol samples using hexane and combinations of hexane with methylene chloride and acetone followed by TOA. The removed OC included different classes of organic compounds based on polarity, e.g., 10% nonpolar, 23% low polar, and 22% polar OC. Despite the great potential, the insolubility method has not been investigated to a wide range of solvent properties [8].

Supercritical fluids, mainly supercritical carbon dioxide (scCO_2_), have gained popularity over the last two decades due to their unique solvent properties. scCO_2_ presents properties between a gas and a liquid, e.g., high diffusivity, near liquid-like density, low surface tension, and low viscosity [33]. One of the main benefits of scCO_2_ lies in its low critical temperature (304.24 K) and high pressure (73.9 bars) that allows removal of organic compounds at mild extraction conditions. Neat scCO_2_ is a non-polar (hexane-like) solvent. In comparison to conventional solvents, the solvent properties of scCO_2_ (polarity and density) can also be fine-tuned by the addition of co-solvents and by controlling temperature and pressure, respectively. Furthermore, the high diffusivity of scCO_2_ leads to increased rates of mass transfer and reduces the extraction time from hours to minutes in comparison to conventional solvent extraction methods [34]. This makes scCO_2_ modified with a co-solvent suitable for the extraction (in a single step) of a wide range of OC fractions, ranging from non-polar to medium polar compounds. Studies on characterization of humic-like substances and organic matter in ambient aerosols demonstrate that up to 70% of organic mass consists of water-insoluble organic matter and neutral humic-like substances with an O/C ratio of 0.1 and 0.4, respectively [35]. The water-insoluble organic matter with an O/C ratio of 0.1 is more likely to undergo pyrolysis in TOA protocols. Non-polar solvents/fluids such as scCO_2_ can best remove these fractions of organic compounds. In the past, scCO_2_ methods have been used for the extraction of different classes of organic compounds from atmospheric aerosols [36–40]. However, to our knowledge, no effort has so far been made to investigate the effects of supercritical fluids for the removal of organic compounds responsible for pyrolysis and for the isolation of EC in atmospheric aerosols. In this study, we aim to investigate the potential of scCO_2_ as well as scCO_2_ with the addition of methanol as co-solvent for the isolation of EC.

## Materials and methods

### Chemicals

Carbon dioxide (99.9993%) with dipped tube (Linde, Sweden), methanol (LC-MS grade, Scharlau, Australia), and MilliQ water (Merck Millipore, Germany) were used for sample pretreatment. For thermo-optical analysis (TOA), helium (99.999%), mixture of methane (99.95%) and helium (99.9996%), hydrogen (99.999%), instrument synthetic air (99.999%), and a mixture of 90% He (99.9996%) and 10% O_2_ (99.9999%) (AGA, Sweden) were used.

### Sampling

Aerosol sampling was conducted on the rooftop of the building of the Department of Physics, Lund University, Lund, Sweden (55° 71′ N, 13° 02′ E, 70 m.a.s.l.). Particulate matter was collected during September and October 2014 with a constant flow rate on circular quartz-fiber filters of a diameter of 102 mm (Pallflex 2500QAT-UP) using a high-volume sampler (TE-5200 Total Suspended Particulate Tri-Pod High Volume Air Sampler, Tisch Environmental). Sampling was performed for 24–72 h to collect samples with low as well as high particulate matter loadings. After sampling, filters were wrapped in aluminum foil and stored in a refrigerator at +5 °C until analysis.

### Supercritical fluid extraction

**Fig. 1:**
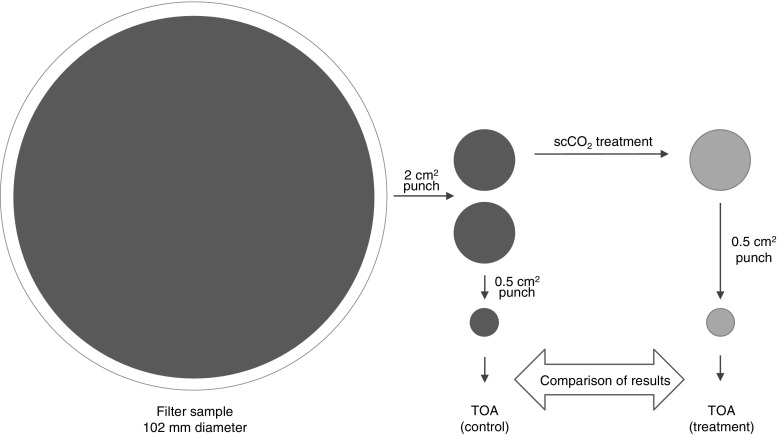
Schematic extraction and TOA procedure for the removal of pyrolytic OC and isolation of EC from OC by supercritical fluid

**Fig. 2:**
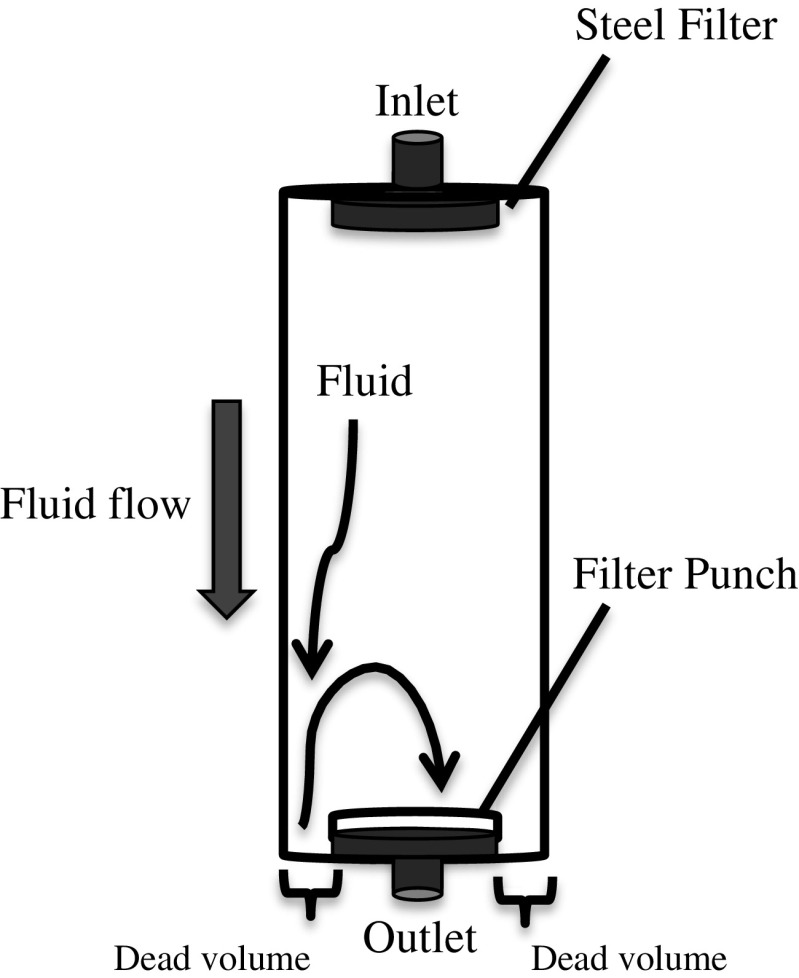
Orientation of filter punch inside scCO_2_ extraction cell. The filter punch is placed before cell outlet in the extraction cell with particulate matter facing towards cell inlet. This orientation allows all the fluid to pass through the filter punch

A circular punch of 2 cm^2^ was used to take sub-samples from the aerosol filters. The schematic of the analysis procedure is given in Fig. 1. Supercritical fluid extractions for OC removal were carried out using a Waters ASFE MV10 system with Waters 5 mL vessels (17-4PH material) for subsequent TOA analysis. The cell was filled up with scCO_2_ at a flow rate of 5 mL/min. When the desired pressure and temperature were reached, the cell was kept pressurized for 20 min. After that, the cell was depressurized and scCO_2_ was allowed to escape from the cell outlet. It was observed that the orientation of filter punches made a large impact on OC removal, EC recovery, and reproducibility of results (Fig. 2 presents the suitable orientation). The filter punches treated with scCO_2_ were analyzed by TOA and the results were compared to untreated controls.

### Screening experiments

**Fig. 3:**
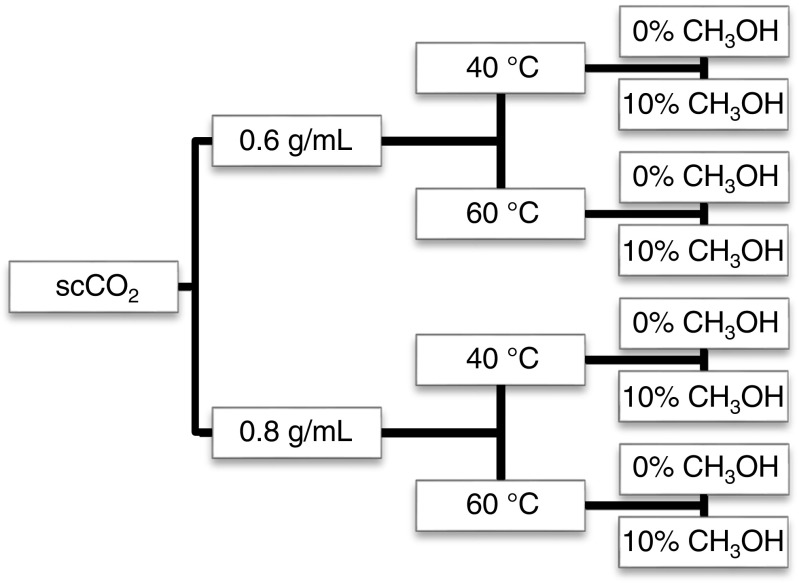
Different conditions of temperature and methanol content with different supercritical carbon dioxide densities used for screening experiments

Screening experiments were carried out to test the solubility behavior of OC at different densities and polarities of scCO_2_ by means of varying extraction temperature, pressure, and addition of methanol, respectively (Fig. 3). Screening experiments were performed with two duplicates to confirm system stability. Only the most suitable conditions for the removal of OC including pyrolytic OC and EC recovery were tested for repeatability.

### Method development

In the light of the screening experiments, the effects of different amounts of methanol as a co-solvent were tested to find the most suitable conditions. Four different compositions of methanol/scCO_2_ systems with 5:95, 10:90, 15:85, and 20:80 (ratios by volume) at 40 °C and 270 bars (other conditions are same as mentioned in “Supercritical fluid extraction” section) were used to test if increasing the amount of methanol helped dissolve more OC present in aerosol samples. In another experiment, the different methanol/scCO_2_ ratios 10:90, 20:80, and 30:70 were tested in a three-step extraction without depressurizing the system in between. A scCO_2_-methanol envelope diagram with extraction conditions for temperature and pressure was derived from literature to achieve supercritical state in all methanol/scCO_2_ ratios. These conditions correspond to 52 °C at 95 bars, 65 °C at 115 bars, and 94 °C at 152 bars, respectively [41, 42].

### Performance of method

scCO_2_ with 10% methanol was tested at various temperatures and pressures to find the most suitable conditions based on maximum OC removal, highest EC recovery, and complete removal of pyrolytic OC. The method was further tested for repeatability using triplicates.

### Comparison with samples treated with water

Samples were treated with water for comparison. Removal of water-soluble organic compounds was performed in three different ways. In the first experiment, 1 mL milliQ water was taken in a 25 mL beaker. A punch of 2 cm^2^ of sample filter was placed in the water with the particulate matter facing up. The sample was sonicated for 15 min (Elmasonic S 30 (H), Elma Schmidbauer, Germany). The filter sample was then removed and dried over silica gel overnight. In the second experiment, 1 mL milliQ water was taken in a 25 mL beaker. A punch of 2 cm^2^ of sample filter was soaked overnight in water with the particulate matter facing up. The samples were dried for up to 20 h on silica gel. In a third experiment, a punch of 2 cm^2^ of sample filter was placed in a 5 mL SS vessel with the particulate matter facing towards the inlet (Fig. 2). MilliQ water was pumped through the sample at a rate of 1 mL/min for 10 min using a standard liquid chromatography pump. Samples were dried over silica gel overnight. The dried samples were kept in a refrigerator at +5 °C wrapped in aluminum foil until thermo-optical analysis.

### Thermo-optical analysis

TOA was conducted on 0.5 cm^2^ punches taken from 2 cm^2^ punches extracted with supercritical fluid and untreated controls (Fig. 1). The punches were analyzed for OC and EC using a DRI Model 2001 OC/EC Carbon Analyzer (Atmoslytic, Calabasas, CA, USA). The EUSAAR_2 protocol was chosen for the quantification and separation of OC, EC, and pyrolytic OC [30]. The EUSAAR_2 protocol is a standardized method used by the European science community and monitoring networks (e.g., EMEP) developed with special emphasis on reducing the charring and pyrolytic OC formation [30]. In the protocol, the filter punch is heated in four steps up to 650 °C in pure helium to evolve OC. The temperature is then decreased to 500 °C, oxygen (10%) is added, and the temperature is ramped up to 850 °C in four steps to evolve EC. To monitor the split between OC and EC, a He/Ne laser beam (633 nm) is continuously attenuated through the filter. The transmission of the laser is monitored and when the transmission reaches its initial value, the carbon left on the filter is determined as EC (instrument detection limit = 0.05 μg/cm^2^, measurement uncertainty = ±0.03 μg/cm^2^).

## Results and discussion

Table 1 shows original filter loadings for untreated samples in terms of OC, pyrolytic OC, EC, and total carbon (TC) estimated by TOA as described in “Thermo-optical analysis” section.

**Table 1:** Flter loadings for OC, EC, pyrolytic OC, and TC on a filter punch of 0.5 cm^2^ of untreated control samples estimated by TOA

Loadings (μg/cm^2^)	Filter 1	Filter 2	Filter 3	Filter 4	Filter 5
OC	32.2	24.6	18.9	35.1	35.6
EC	8.9	8.2	5.7	20.9	12.2
Pyrolytic OC	3.2	1.9	1.0	2.1	2.2
TC	41.2	32.8	24.5	56.0	47.8

### Screening experiments

The screening experiments showed that scCO_2_ with 10% methanol by volume as a co-solvent was suitable for the removal of measurable pyrolytic OC from aerosol samples as compared to neat scCO_2_ since 49–57% OC was removed with 81–88% EC recovery when 10% methanol was added (Table 1 shows filter loadings as filter 1). Table 2 gives details of extraction conditions, measured OC, EC, pyrolytic OC values, percentage OC removal, and percentage EC recovery at the eight different treatment conditions applied on filter 1. Percentage OC removal and EC recovery were calculated by Eqs. (1) and (2), respectively.

1 $$ \mathrm{OC}\ \mathrm{removal}\ \left(\%\right)=\left({\mathrm{OC}}_{\mathrm{control}}-{\mathrm{OC}}_{\mathrm{sample}}\right)\times 100/{\mathrm{OC}}_{\mathrm{control}} $$

2 $$ \mathrm{EC}\ \mathrm{recovery}\ \left(\%\right)={\mathrm{EC}}_{\mathrm{sample}}\times 100/{\mathrm{EC}}_{\mathrm{control}} $$

**Table 2:** Screening experiments showing the removal of OC and recovery of EC treating filter 1 with supercritical carbon dioxide at various conditions, relative standard deviations estimated on duplicates

Treatment	Treatment conditions				Amounts of carbonaceous fractions estimated by TOA (μg/cm^2^)			OC removal (%)	EC recovery (%)
	Temperature (°C)	Pressure (bar)	Density of scCO_2_ (g/mL)	CH_3_OH (%)	OC	EC	Pyrolytic OC		
1	40	98	0.6	0	20.9	9.2	–	35	103^a^
2	60	150	0.6	0	23.8 ± 3.2	7.2 ± 3.1	2.1	26 ± 10	81 ± 35
3	40	270	0.8	0	24.7	6.2	3.1	23	69
4	60	350	0.8	0	28.3	6.2	5.0	12	69
5	40	98	0.6	10	17.1 ± 0.9	7.2 ± 0.3	–	47 ± 03	81 ± 03
6	60	150	0.6	10	16.4	7.3	–	49	82
7	40	270	0.8	10	15.2 ± 2.2	7.8 ± 0.42	–	53 ± 07	88 ± 05
8	60	350	0.8	10	13.9	7.4	–	57	82

^a^Untreated control pyrolyzed giving rise to less true EC as compared to the sample leading to an EC recovery of above 100%. Standard deviation is high enough that it may also be considered as EC recovery close to 100%

No significant effect of change in scCO_2_ density or temperature on OC removal was observed. The density of scCO_2_ affects its solvent properties in terms of diffusivity that allows scCO_2_ to penetrate in the small pores of multiple layers of sample matrix and dissolve the analyte. The mass of particulate matter deposited on filters is usually very small, i.e., ∼μg, making up a very thin layer of particulate matter. Hence, low density of scCO_2_ was good enough to remove a significant fraction of OC in aerosol samples. The removal of 49–57% OC using scCO_2_ with addition of 10% methanol shows that the aerosol samples used in the experiments may contain up to 57% non-polar to medium polar organic compounds. The removed fraction may include organic compounds with low molecular masses, e.g., carboxylic acids, anhydrous sugars, methoxyphenols, and other similar classes of organic compounds as reported in earlier studies [35]. No characterization of extracted organic compounds was performed. Matrix effects, method kinetics, and influence of different fluid flow profiles were not investigated in this study.

### Method development

The screening experiments showed that the addition of methanol as co-solvent to scCO_2_ had an impact on the removal of OC. The effect of the methanol contents, i.e., 5, 10, 15, and 20% (*v*/*v*), was tested at 40 °C and 270 bars using subsamples taken from filter 2 (Table 1). Percentage OC removal and EC recoveries were 48 and 62% with 5% methanol; 49 and 64% with 10% methanol; 44 and 51% with 15% methanol, and 47 and 60% with 20% methanol (percent difference 12 and 23%, *n* = 2), respectively. The results indicate that nearly 50% of the extractable OC in the samples contained low-medium polar compounds due to the fact that scCO_2_ with small amounts of methanol cannot dissolve highly polar compounds. Filter 3 was used for a three-step extraction with different amounts of methanol (10, 20, 30% all at supercritical state) as co-solvent without depressurizing the system in between (*n* = 2). The extraction method did not show significant OC removal (i.e., up to 25%) in comparison to when a methanol/scCO_2_ ratio of 10:90 by volume was used. However, no pyrolytic OC was observed in these experiments. It was observed that such harsh extraction conditions led to poor EC recoveries, i.e., 55%. It is not known at the moment whether the poor EC recoveries are due to partial solubility of larger organic molecules in scCO_2_, which behave as EC in TOA [12, 13], alternatively if some of the EC flushes away from filter mechanically.

In the present study, the 90:10% scCO_2_-methanol mixture was found to be the most suitable for the removal of a significant fraction of OC (>50%) including the compounds responsible for pyrolysis in ambient aerosol samples. Based on the solvent properties of aforementioned supercritical fluid, these findings are in agreement with that of Chen et al. [35] reporting the presence of water-insoluble organic compounds and neutral humic-like substances possessing O/C ratios of 0.1 and 0.4, respectively, as up to 70% of total organic mass in ambient aerosol samples. Previous studies have also shown that a scCO_2_-methanol system (90:10%) was capable of extracting 36–45% humic acid from archeological samples [43]. It is yet to be discovered if rest of the OC contains polar organic compounds insoluble in scCO_2_-methanol mixture and/or large organic molecules that oxidize in TOA but are prone to solubility on mild conditions. Based on the study of Rowe et al. [43], it may be concluded that polar humic-like substances do not get dissolved in scCO_2_-methanol system but respond to TOA protocols as OC.

### Comparison with samples treated with water

Ultrasonic-assisted water extraction gave an EC recovery of 37 ± 5%. The high energy of the ultrasonic waves may have caused particulate matter to flush away from the filter resulting in lower EC recovery compared to samples treated by soaking in water and pumping water through the filters. Table 3 gives a comparison in OC removal and EC recovery of treatments using water and scCO_2_ with 10% methanol. The samples treated with water showed lower EC recovery in comparison to the literature [30, 31]. The lower EC recovery may be related to the nature of the samples, i.e., presence of water insoluble substances and mechanical loss of sampled particles from the filters possibly due turbulence in the flow of water caused by the dead volume of the extraction cell (Fig. 2).

**Table 3:** Comparison of treatments using water and scCO_2_ with 10% methanol (treatment 7 in Table 2), standard deviations estimated on duplicates for treatment 1 and triplicates for the rest

No.	Treatment	Filter no.	OC removal (%)	EC recovery (%)
1	scCO_2_ at 40 °C and 270 bars (*ρ* = 0.6 g/mL), with 10% methanol	1	53 ± 7	88 ± 5
2	Ultrasonication with water	4	31 ± 7	37 ± 5
3	Soaking in water	4	38 ± 3	38 ± 4
4	Water pumping through filter	4	42 ± 1	38 ± 4

### Performance of method

The experiments in “Method development” section reveal that the use of 10% methanol by volume as co-solvent to scCO_2_ was the most suitable combination. In this context, triplicates were taken to test performance of the method. The best experimental conditions found were methanol/scCO_2_ ratio of 10:90 by volume at a temperature of 60 °C, pressure 350 bars for complete removal of measurable pyrolytic OC, 59 ± 3% OC removal, and 78 ± 16% EC recovery using subsamples taken from filter 5. It was found that one out of three replicates slightly deviated leading to a high relative standard deviation of percentage EC recovery. A non-uniform distribution of particulate matter over the filter may be one of the reasons. In comparison to other wet sample pretreatment methods, the scCO_2_-methanol method is faster (i.e., 20 min) for the removal of OC including pyrolytic OC. The potential of the technique can be explored with more knowledge of chemical structures of different compounds constituting OC. The EC recovery (≈80%) was also comparable to other thermal and wet methods [28, 9, 29].

## Conclusion and outlook

A sample pretreatment method using supercritical CO_2_ was developed for the isolation and estimation of EC by TOA. The method was applicable for the removal of ≈60% OC including pyrolytic OC while retaining a large fraction of EC (i.e., ≈80% EC recovery) in ambient aerosol samples. Use of scCO_2_ with and without methanol as co-solvent is environmentally green and the performance of the method is higher than other wet methods in terms of time, percentage removal of OC including pyrolytic OC and percentage EC recovery. The method was successfully applied to ambient aerosol samples. The method needs to be tested for source-specific samples, e.g., samples from combustion of biomass and fossil fuels. The beauty of scCO_2_ method lies in the fact that the sample pretreatment method can be fine-tuned by means of mild temperature, density and co-solvent for unique samples collected from certain sources of emissions.

Due to the molecular complexity of OC and the effects of emissions and atmospheric chemical transformation on chemical composition of atmospheric aerosols, an ideal solvent/fluid should dissolve different classes of organic compounds with different functional groups and polarities. Since none of the known solvent/fluid possesses such dramatic properties, complete isolation of EC has not been achieved by insolubility so far. In this connection, a two-step method for the isolation of EC from OC is proposed by coupling the scCO_2_ sample pretreatment to thermal treatment like other contemporary methods. After the removal of ≈60% OC from ambient aerosols samples by scCO_2_ and 10% methanol, the remaining OC can be combusted by TOA protocols with no interfering pyrolytic OC. Once all the measureable OC is removed, the TOA protocol can then be stopped and the obtained EC can be used for ^14^C analysis. The proposed method has potential to be used prior to ^14^C analysis of ambient aerosol samples (to avoid pyrolytic OC to be analyzed as EC). Although, due to the fact that scCO_2_ turns into its gaseous form by releasing pressure (at the end of the process) which minimizes the risk of contamination, further studies are needed to test the applicability of the method and whether the method contaminates the recovered EC with carbon from scCO_2_ and/or methanol or not. The method is presented as a proof of concept with potential of fine-tuning the solvent properties that can be used for pyrolytic OC free sample preparation and for the estimation and isolation of EC.

## References

[1] WHO. WHO. The world health report 2002—reducing risks, promoting healthy life. WHO; 2002. 10.1080/1357628031000116808 14741909

[2] WHO. WHO. Health aspects of air pollution. Copenhagen, Denmark. 2004.

[3] Solomon S, Qin D, Manning M, Chen Z, Marquis M, Averyt KB et al. Contribution of Working Group I to the Fourth Assessment Report of the Inter-governmental Panel on Climate Change. Cambridge University Press; 2007a.

[4] Hyder M, Genberg J, Sandahl M, Swietlicki E, Jonsson JA (2012). Yearly trend of dicarboxylic acids in organic aerosols from south of Sweden and source attribution. Atmos Environ.

[5] Fuzzi S, Baltensperger U, Carslaw K, Decesari S, van Der Gon HD, Facchini MC (2015). Particulate matter, air quality and climate: lessons learned and future needs. Atmos Chem Phys.

[6] Genberg J, Hyder M, Stenstrom K, Bergstrom R, Simpson D, Fors EO (2011). Source apportionment of carbonaceous aerosol in southern Sweden. Atmos Chem Phys.

[7] Petzold A, Ogren JA, Fiebig M, Laj P, Li SM, Baltensperger U (2013). Recommendations for reporting “black carbon” measurements. Atmos Chem Phys.

[8] Lack DA, Moosmuller H, McMeeking GR, Chakrabarty RK, Baumgardner D (2014). Characterizing elemental, equivalent black, and refractory black carbon aerosol particles: a review of techniques, their limitations and uncertainties. Anal Bioanal Chem.

[9] Dusek U, Monaco M, Prokopiou M, Gongriep F, Hitzenberger R, Meijer HAJ (2014). Evaluation of a two-step thermal method for separating organic and elemental carbon for radiocarbon analysis. Atmos Meas Tech.

[10] Gustafsson O, Bucheli TD, Kukulska Z, Andersson M, Largeau C, Rouzaud JN (2001). Evaluation of a protocol for the quantification of black carbon in sediments. Global Biogeochem Cy.

[11] Hammes K, Schmidt MWI, Smernik RJ, Currie LA, Ball WP, Nguyen TH et al. Comparison of quantification methods to measure fire-derived (black/elemental) carbon in soils and sediments using reference materials from soil, water, sediment and the atmosphere. Glob Biogeochem Cycle. 2007;21(3). doi:10.1029/2006gb002914.

[12] Andreae MO, Gelencser A (2006). Black carbon or brown carbon? The nature of light-absorbing carbonaceous aerosols. Atmos Chem Phys.

[13] Chang SG, Brodzinsky R, Gundel LA, Novakov T, Wolff GT, Klimisch RL (1982). Chemical and catalytic properties of elemental carbon. Particulate carbon: atmospheric life cycle.

[14] Dusek U, ten Brink HM, Meijer HAJ, Kos G, Mrozek D, Rockmann T (2013). The contribution of fossil sources to the organic aerosol in the Netherlands. Atmos Environ.

[15] Currie LA (2000). Evolution and multidisciplinary frontiers of C-14 aerosol science. Radiocarbon.

[16] El Haddad I, Marchand N, Wortham H, Piot C, Besombes JL, Cozic J (2011). Primary sources of PM2.5 organic aerosol in an industrial Mediterranean city, Marseille. Atmos Chem Phys.

[17] Gelencser A, May B, Simpson D, Sanchez-Ochoa A, Kasper-Giebl A, Puxbaum H et al. Source apportionment of PM2.5 organic aerosol over Europe: Primary/secondary, natural/anthropogenic, and fossil/biogenic origin. J Geophys Res Atmos. 2007;112(D23). doi:10.1029/2006jd008094.

[18] Gilardoni S, Vignati E, Cavalli F, Putaud JP, Larsen BR, Karl M (2011). Better constraints on sources of carbonaceous aerosols using a combined C-14 - macro tracer analysis in a European rural background site. Atmos Chem Phys.

[19] Glasius M, la Cour A, Lohse C. Fossil and nonfossil carbon in fine particulate matter: a study of five European cities. J Geophys Res Atmos. 2011;116. doi:10.1029/2011jd015646.

[20] Schichtel BA, Malm WC, Bench G, Fallon S, McDade CE, Chow JC et al. Fossil and contemporary fine particulate carbon fractions at 12 rural and urban sites in the United States. J Geophys Res Atmos. 2008;113(D2). doi:10.1029/2007jd008605.

[21] Szidat S, Jenk TM, Synal HA, Kalberer M, Wacker L, Hajdas I et al. Contributions of fossil fuel, biomass-burning, and biogenic emissions to carbonaceous aerosols in Zurich as traced by C-14. J Geophys Res Atmos. 2006;111(D7). doi:10.1029/2005jd006590.

[22] Szidat S, Prevot ASH, Sandradewi J, Alfarra MR, Synal HA, Wacker L et al. Dominant impact of residential wood burning on particulate matter in Alpine valleys during winter. Geophys Res Lett. 2007;34(5). doi:10.1029/2006gl028325.

[23] Szidat S, Ruff M, Perron N, Wacker L, Synal HA, Hallquist M (2009). Fossil and non-fossil sources of organic carbon (OC) and elemental carbon (EC) in Goteborg. Sweden Atmos Chem Phys.

[24] Moosmuller H, Chakrabarty RK, Arnott WP (2009). Aerosol light absorption and its measurement: a review. J Quant Spectrosc Ra.

[25] Raman CV, Krishnan KS (1928). A new type of secondary radiation. Nature.

[26] Skoog DA, Holler FJ, Crouch SR. Principles of instrumental analysis. (6th ed.) Cengage Learning; 2006.

[27] Willard HH, Meritt LL, Dean JJ, Settle FA (1988). Instrumental methods of analysis.

[28] Bernardoni V, Calzolai G, Chiari M, Fedi M, Lucarelli F, Nava S (2013). Radiocarbon analysis on organic and elemental carbon in aerosol samples and source apportionment at an urban site in Northern Italy. J Aerosol Sci.

[29] Zhang YL, Perron N, Ciobanu VG, Zotter P, Minguillon MC, Wacker L (2012). On the isolation of OC and EC and the optimal strategy of radiocarbon-based source apportionment of carbonaceous aerosols. Atmos Chem Phys.

[30] Cavalli F, Viana M, Yttri KE, Genberg J, Putaud JP (2010). Toward a standardised thermal-optical protocol for measuring atmospheric organic and elemental carbon: the EUSAAR protocol. Atmos Meas Tech.

[31] Piazzalunga A, Bernardoni V, Fermo P, Valli G, Vecchi R (2011). Technical note: on the effect of water-soluble compounds removal on EC quantification by TOT analysis in urban aerosol samples. Atmos Chem Phys.

[32] Cheng Y, He KB, Duan FK, Du ZY, Zheng M, Ma YL (2012). Characterization of carbonaceous aerosol by the stepwise-extraction thermal-optical-transmittance (SE-TOT) method. Atmos Environ.

[33] Beckman EJ. Supercritical and near-critical CO2 in green chemical synthesis and processing. J Supercrit Fluids. 2004;28.

[34] Jumaah F. Extraction and chromatographic separation of lipids and lipid-like compounds using compressed carbondioxide as a solvent. Lund University; 2016.

[35] Chen Q, Ikemori F, Higo H, Asakawa D, Mochida M (2016). Chemical structural characteristics of HULIS and other fractionated organic matter in urban aerosols: results from mass spectral and FT-IR analysis. Environ Sci Technol.

[36] Lian JJ, Ren Y, Chen JM, Wang T, Cheng TT (2009). Distribution and source of alkyl polycyclic aromatic hydrocarbons in dustfall in Shanghai, China: the effect on the coastal area. J Environ Monitor.

[37] Chiappini L, Perraudin E, Durand-Jolibois R, Doussin JF (2006). Development of a supercritical fluid extraction-gas chromatography-mass spectrometry method for the identification of highly polar compounds in secondary organic aerosols formed from biogenic hydrocarbons in smog chamber experiments. Anal Bioanal Chem.

[38] Shimmo M, Jantti J, Aalto P, Hartonen K, Hyotylainen T, Kulmala M (2004). Characterisation of organic compounds in aerosol particles from a Finnish forest by on-line coupled supercritical fluid extraction-liquid chromatography-gas chromatography-mass spectrometry. Anal Bioanal Chem.

[39] Shimmo M, Adler H, Hyotylainen T, Hartonen K, Kulmala M, Riekkola ML (2002). Analysis of particulate polycyclic aromatic hydrocarbons by on-line coupled supercritical fluid extraction-liquid chromatography-gas chromatography-mass spectrometry. Atmos Environ.

[40] Lang QY, Hunt F, Wai CM (2000). Supercritical fluid extraction of polycyclic aromatic hydrocarbons from white pine (*Pinus strobus*) needles and its implications. J Environ Monitor.

[41] Leu AD, Chung SYK, Robinson DB (1991). The equilibrium phase properties of (carbon-dioxide + methanol). J Chem Thermodyn.

[42] Yeo SD, Park SJ, Kim JW, Kim JC (2000). Critical properties of carbon dioxide + methanol, + ethanol,+1-propanol, and+1-butanol. J Chem Eng Data.

[43] Rowe MW, Phomakay J, Lay JO, Guevara O, Srinivas K, Hollis WK (2013). Application of supercritical carbon dioxide-co-solvent mixtures for removal of organic material from archeological artifacts for radiocarbon dating. J Supercrit Fluid.

